# Supplementary material for: The effects of host species and sexual dimorphism differ among root, leaf and flower microbiomes of wild strawberries in situ
Source: Sci Rep. 2018 Mar 26;8:5195. doi: 10.1038/s41598-018-23518-9 (PMC5979953; doi:10.1038/s41598-018-23518-9)
Supplement: Supplementary file 1 — Figures S1–S7 [file 41598_2018_23518_MOESM1_ESM.pdf]

Supplementary Material

The effects of host species and sexual dimorphism differ among root, leaf and flower microbiomes of wild strawberries *in situ*

Na Wei[*]**, Tia-Lynn Ashman**[*]

[*]**Correspondence**:
Na Wei (Email: na.wei@pitt.edu)
Tia-Lynn Ashman (Email: tia1@pitt.edu)

Table of Contents:

Figure S1. OTU overlap among root, leaf and flower microbiome in each host species. Page 2

Figure S2. PCoAs of phylogenetic β-diversity metrics revealing microbial community separation primarily driven by organ type rather than host species. Page 3

Figure S3. Differentially abundant OTUs in flower and leaf relative to root microbiomes. Page 4

Figure S4. Overlaps in differentially abundant OTUs identified using different methods. Page 5

Figure S5. Constrained PCoAs of weighted betaMPD revealing microbial community clustering among host species varying in roots, leaves and flowers. Page 6

Figure S6. Sexual dimorphism in microbial α-diversity detected in flower microbiomes. Page 7

Figure S7. Sexual dimorphism in relative abundances of bacterial phyla in leaf microbiomes. Page 8

Figure S1. OTU overlap among root, leaf and flower microbiome in each host species. These three host species are (**a**) *F. chiloensis* (F.chilo), (**b**) *F.* ×*ananassa* ssp. *cuneifolia* (F.cunei), and (**c**) *F. virginiana* ssp. *platypetala* (F.virg).

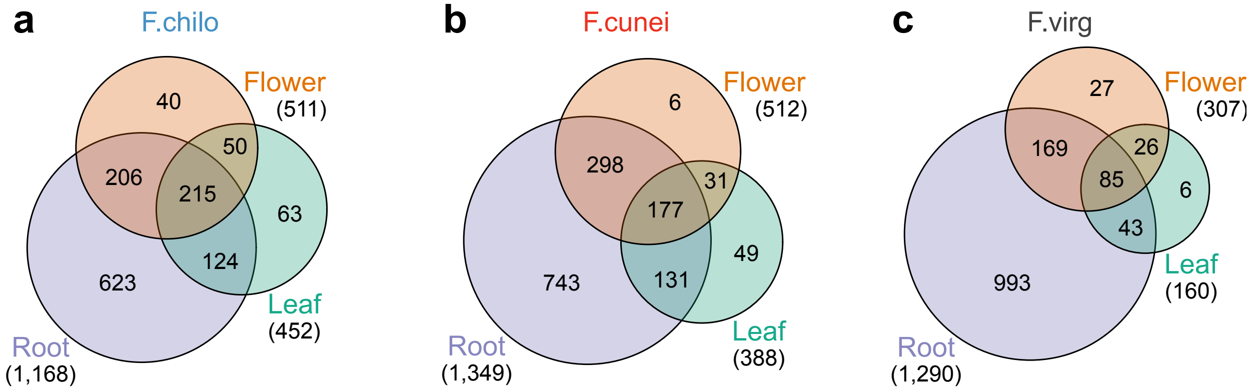

Figure S2. PCoAs of phylogenetic β-diversity metrics revealing microbial community separation primarily driven by organ type rather than host species. (**a**) PCoA of abundance-weighted UniFrac distance, in which UniFrac distance is defined as the portion of branch lengths of OTUs not shared between two communities, weighted by abundances. (**b**) PCoA of abundance-weighted betaMPD distance, in which betaMPD represents mean pairwise branch lengths of OTUs between two communities, weighted by abundances. In both panels, the ellipses (based on 2 s.d.) are indicative of the spread of microbial communities within each organ type.

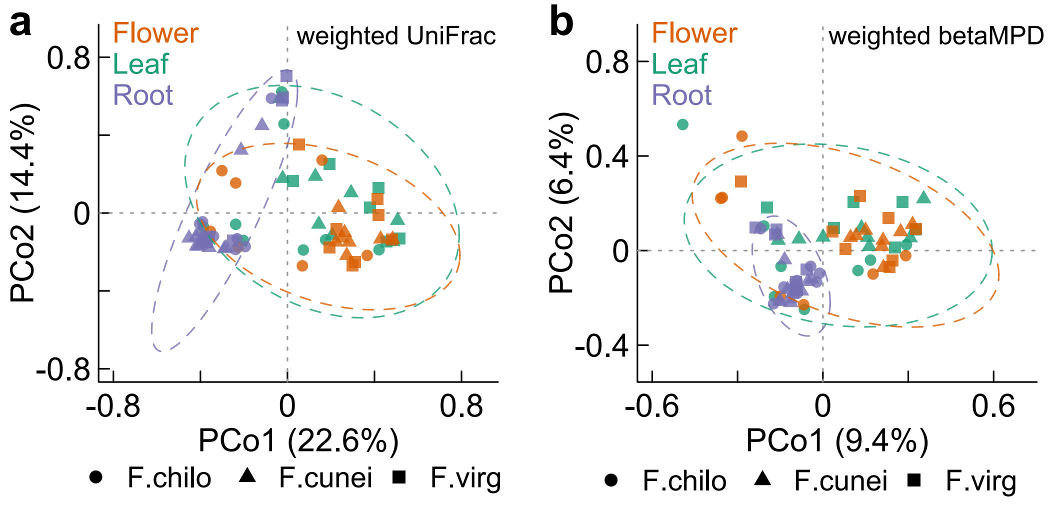

Figure S3. Differentially abundant OTUs in flower (a) and leaf (b) relative to root microbiomes. (**a**) Significantly enriched and depleted OTUs in flower relative to root microbiomes are shown as large, dark gray dots, identified by FDR-GLMs. OTUs with non-significant abundance changes are shown as small, light gray dots. The orange and purple dots of the immediate point size denote significant OTUs of differential abundances in response to organ type, identified by FWER-GLMs. (**b**) FDR-GLMs were also conducted to identity enriched and depleted OTUs in leaf relative to root microbiomes. The green and purple dots denote the OTUs responding significantly to organ type, identified by FWER-GLMs. In both panels, bacterial phyla and individual OTUs within each phylum are ordered along the *x*-axis by their relative abundances in the whole data set. Dashed horizontal lines depict (log_2) fold change of 2 and 5 (as well as -2 and -5).

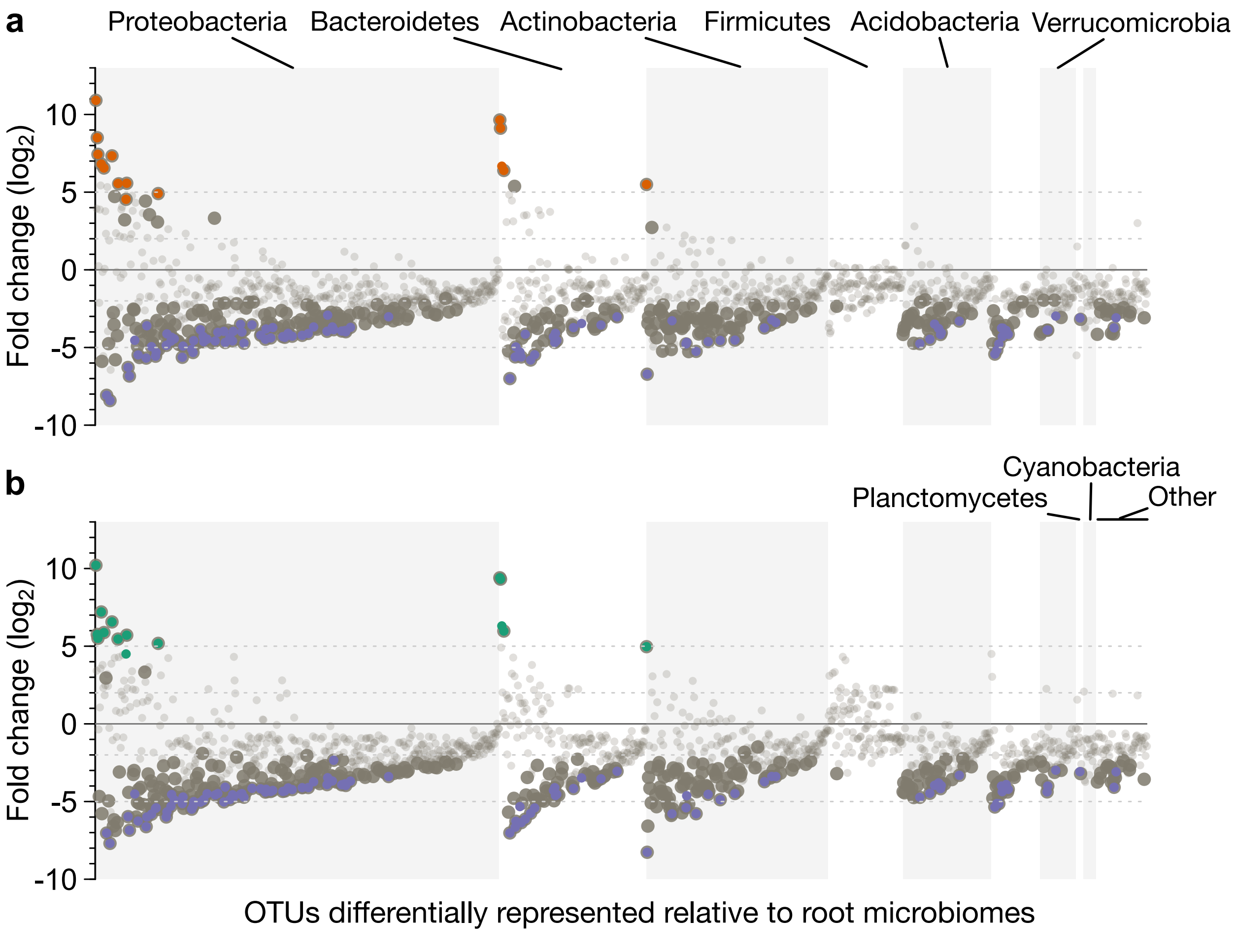

Figure S4. Overlaps in differentially abundant OTUs identified using different methods. GLMs with negative binomial errors in mvabund[1] use stringent resampling-based control of family-wise error rate (FWER), which also consider dependence structure among OTUs. GLMs with negative binomial errors in edgeR[2] use false discovery rate (FDR) control for multiple testing. FWER-GLMs in mvabund identified 120 OTUs as significantly responding to organ type. FDR-GLMs in edgeR identified 404 OTUs of differential abundances between flower and root microbiomes, and 414 OTUs between leaf and root microbiomes.

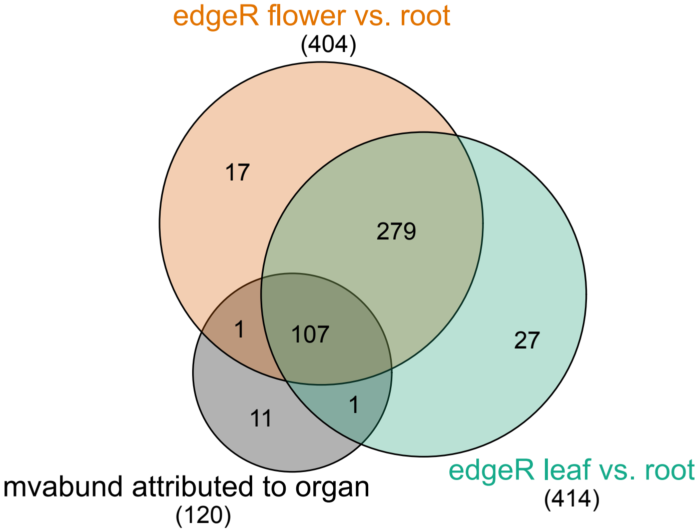

Figure S5. Constrained PCoAs of weighted betaMPD revealing microbial community clustering among host species varying in roots, leaves and flowers. Weighted betaMPD is a phylogenetic β-diversity metric that is insensitive to species richness. It's defined as mean pairwise branch lengths of OTUs between two microbial communities, weighted by abundances. Constrained PCoAs here were controlled for abiotic environments (PC1.clim and PC2.clim) and host sex (female and male/hermaphrodite).

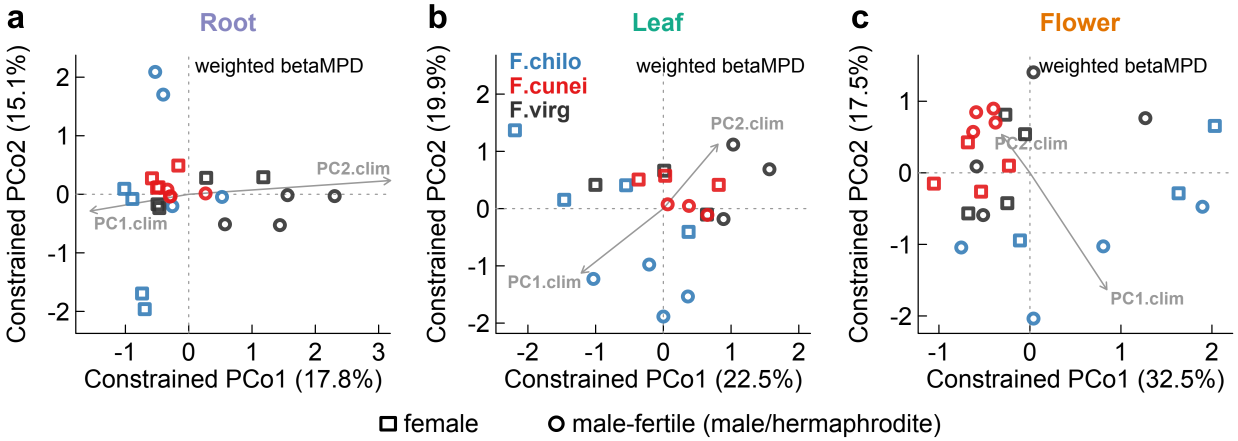

Figure S6. Sexual dimorphism in microbial α-diversity detected in flower microbiomes. The effects of host sex and its interaction with host plant species on microbial α-diversity were modeled using general linear models (LMs; Table 1), controlling for abiotic environments. The least-squares means (LS-means) and error bars (1 s.e.m.) were plotted for each host plant species and sex (females, squares; males or hermaphrodites, circles). Only significant LS-mean difference between sex is indicated: *, $P \leq 0.05$.

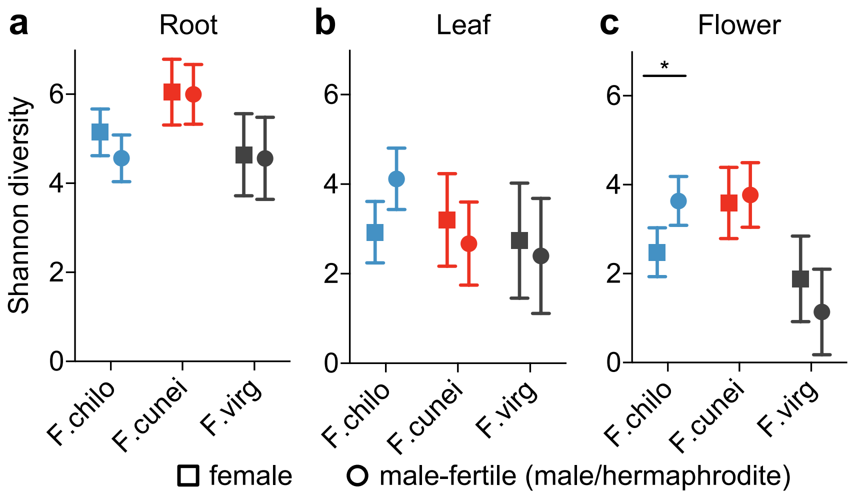

Figure S7. Sexual dimorphism in relative abundances of dominant bacterial phyla in leaf microbiomes. Statistical significance was assessed using proportion tests with false discovery rate control for multiple testing (alpha = 0.05): ***, $P \leq 0.001$.

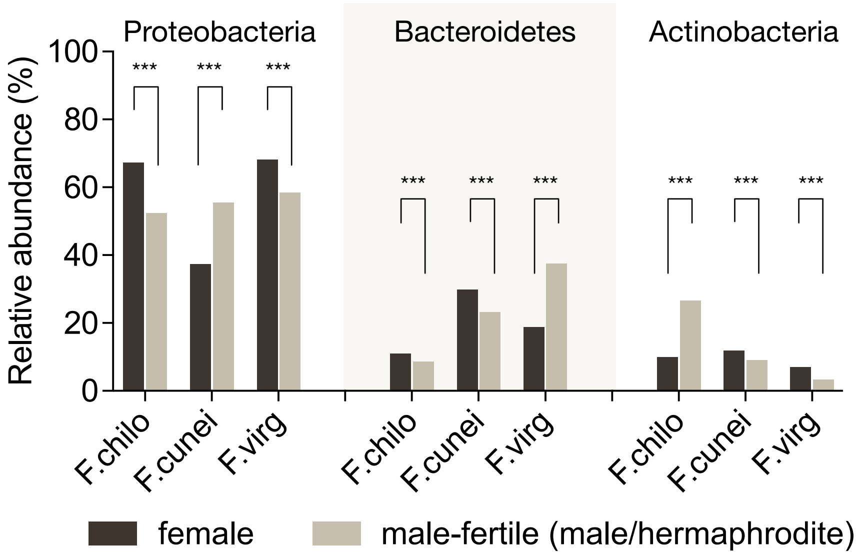

References

1 Wang, Y., Naumann, U., Wright, S. T. & Warton, D. I. mvabund – an R package for model-based analysis of multivariate abundance data. *Methods Ecol. Evol.* **3**, 471-474 (2012).

2 Robinson, M. D., McCarthy, D. J. & Smyth, G. K. edgeR: a Bioconductor package for differential expression analysis of digital gene expression data. *Bioinformatics* **26**, 139-140 (2010).
